# Transcriptional insights of citrus defense response against *Diaporthe citri*

**DOI:** 10.1186/s12870-023-04624-x

**Published:** 2023-12-04

**Authors:** Pudong Li, Xiaoe Xiao, Jingrui Wang, Fan Niu, Jiangnan Huang, Bianyue Xie, Lu Ye, Chaofan Zhang, Dengliang Wang, Qun Wu, Xueliang Zheng, Yunpeng Gai, Hongye Li, Chen Jiao

**Affiliations:** 1 The Key Laboratory of Molecular Biology of Crop Pathogens and Insects of Ministry of Agriculture, The Key Laboratory of Biology of Crop Pathogens and Insects of Zhejiang Province, Institute of Biotechnology, Zhejiang University, Hangzhou, 310058 Zhejiang China; 2 Quzhou Academy of agricultural and Forestry Sciences, Quzhou, 323000 Zhejiang China; 3 Agricultural Characteristic Industry Development Center of Quzhou City, Quzhou, Zhejiang 323000 China; 4 School of Grassland Science, Beijing Forestry University, Beijing, 100083 China

**Keywords:** Citrus, Melanose Disease, *Diaporthe citri*, RNA-seq, Defense reaction

## Abstract

Citrus melanose, caused by *Diaporthe citri*, is one of the most important and widespread fungal diseases of citrus. Previous studies demonstrated that the citrus host was able to trigger the defense response to restrict the spread of *D*. *citri*. However, the molecular mechanism underlying this defense response has yet to be elucidated. Here, we used RNA-Seq to explore the gene expression pattern at the early (3 days post infection, dpi) and late (14 dpi) infection stages of citrus leaves in response to *D*. *citri* infection, and outlined the differences in transcriptional regulation associated with defense responses. The functional enrichment analysis indicated that the plant cell wall biogenesis was significantly induced at the early infection stage, while the callose deposition response was more active at the late infection stage. CYP83B1 genes of the cytochrome P450 family were extensively induced in the callus deposition-mediated defense response. Remarkably, the gene encoding pectin methylesterase showed the highest upregulation and was only found to be differentially expressed at the late infection stage. Genes involved in the synthesis and regulation of phytoalexin coumarin were effectively activated. F6’H1 and S8H, encoding key enzymes in the biosynthesis of coumarins and their derivatives, were more strongly expressed at the late infection stage than at the early infection stage. Collectively, our study profiled the response pattern of citrus leaves against *D*. *citri* infection and provided the transcriptional evidence to support the defense mechanism.

**Supplementary Information:**

The online version contains supplementary material available at 10.1186/s12870-023-04624-x.

## Background

Citrus is one of the most popular fruits in the world, due to their unique and refreshing flavor, as well as their rich nutrients. Nowadays, citrus is widely grown in over 140 countries throughout the tropical and subtropical regions [1]. The global citrus production in 2019 is over 157 million tons accounting for the largest single category of fruit production in the world (FAO statistics, 2019).

*Diaporthe citri* is one of the most destructive fungal pathogens of citrus [2, 3]. It infects young leaves, shoots and fruits, and induces black-to-reddish brown, raised pustules (called melanose) on the leaves, twigs, and fruits of citrus [4]. Usually, melanose does not reduce yield but impacts the marketability of citrus fruits, leading to heavy economic losses [2, 5]. *D*. *citri* also causes stem-end rot, shoot-blight and dieback, trunk or branch gummosis and rot of all citrus species or varieties worldwide [2, 3, 6–8]. *D*. *citri* can live as endophyte, saprophyte or parasite on citrus [3, 8, 9], however, in most time of the life cycle it grows and produces conidia and ascospores on dead wood of citrus [2, 4]. Under humid conditions, the conidia extrude from pycnidia in slimy masses or tendrils, and are dispersed by water (rain or overhead irrigation) to susceptible tissues in the citrus canopy [2, 4]. The ascospores produced from spherical perithecia are grown in dead wood and are dispersed by wind over longer distance [2, 4].

When spores of *D*. *citri* drop on citrus leaves, they can germinate and penetrate through the cuticle and interact with the epidermal cells, which in turn activates the host defense response, by which the hyphae are restricted in plant cells [10]. The phytoalexin 6, 7-dimethoxy coumarin and cell-division inducer gamma-amino-n-butyric acid (GABA) secreted by citrus cells comprise the mechanical barrier to defend against *D. citri* invasion [11, 12]. The barrier is formed at the damage site, consisting of necrotic cells, callus and periderm [10]. Despite the microscopic evidence of host defense response, the molecular mechanisms on how host genes are organized to counteract fungal invasion are largely elusive.

In the present study, transcriptomes of the mandarin (*Citrus reticulata* cv. Hongjv) leaves inoculated with *D. citri* were profiled at 3 and 14 days post inoculation (dpi). This dataset provides a unique opportunity for investigating transcriptional regulation of citrus in response to *D. citri* invasion.

## Results

### RNA-Seq analysis of *D. citri*-inoculated versus mock-inoculated citrus leaves

**Fig. 1:**
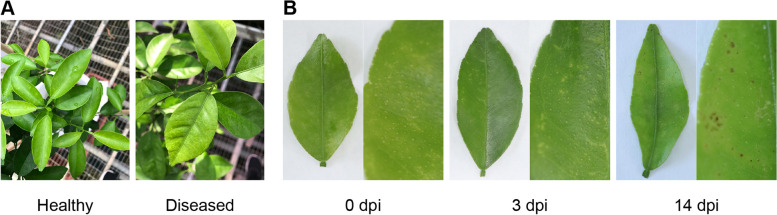
Disease development in citrus leaves infected by *Diaporthe citri*. **A** The symptom of *Citrus reticulata* cv. Hongjv infected by *D*. *citri* in the glasshouse at 14 dpi. **B** Pictures of *D*. *citri* infection progress in citrus leaves up to 14 dpi (days post inoculation)

In the glasshouse, we inoculated citrus (*Citrus reticulata* cv. Hongjv) leaves with conidia of *D*. *citri* (Dc) (Fig. 1A). At 3 dpi, obvious early disease symptoms were observed, with the leaves showing chlorosis spots. At 14 dpi, the leaves developed visible melanose symptoms, with black spot-like lesions (Fig. 1B), and the symptoms stabilized thereafter. Based on the disease symptoms, the infection process of *D. citri* was tentatively defined as early (3 dpi) and late (14 dpi) infection stages. To characterize gene expression dynamics during *D. citri* infection, we performed transcriptome sequencing of Dc-inoculated or mocked citrus leaves at both early and late stages, with each of five biological replicates.

**Fig. 2:**
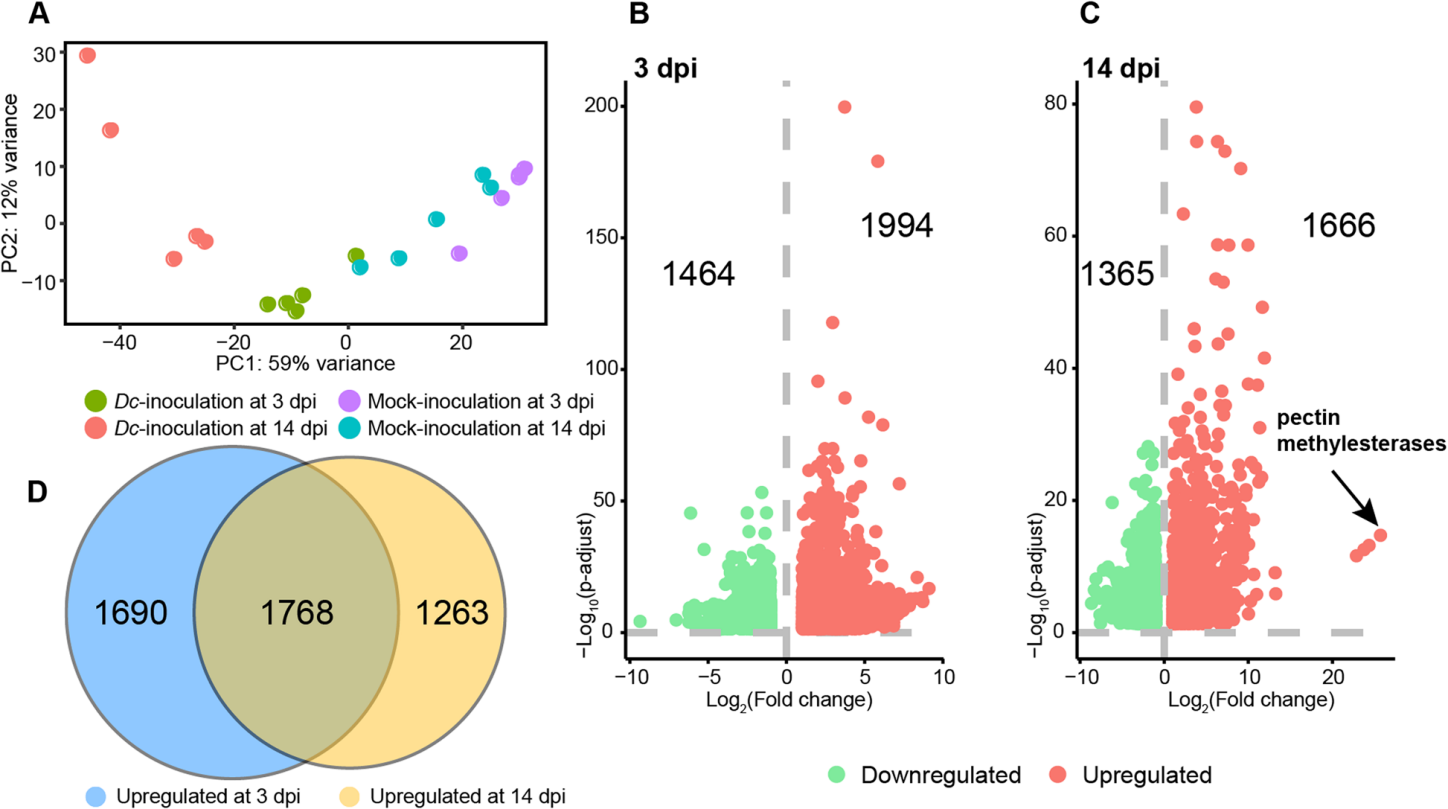
Overview of differential gene expression of citrus leaves infected by *D. citri*. **A** Principal component analysis (PCA) of citrus leaves at different time points with or without Dc infection. **B**, **C** Volcano plots summarizing differentially expressed genes at 3 and 14 dpi, respectively. Each dot represents an upregulated or downregulated gene. **D** Venn diagrams representing common and distinct differentially expressed genes between 3 and 14 dpi

The percentage of reads of all the samples that aligned to the citrus reference genome was 97.0–97.7% (Table S1). A total of 16,896 genes (representing 60.1% of the total number of annotated genes) were detected under the threshold of 1 CPM (count per million) in at least half of the samples. Concerning the pathogen transcriptome, the number of reads mapped to the *D*. *citri* genome [13] from the harvested samples was very low (< 0.01%), prohibiting further analysis of fungal transcriptomes. Principal component analysis separated samples into four groups, reflecting the difference of development stages and fungal invasion (Fig. 2A). Differentially expressed genes between Dc-inoculated and mocked samples at each time point were identified (adjusted *P* < 0.05 and fold change > 2) and listed in Table S2. In general, more upregulated genes than downregulated genes were observed at both time points. Specifically, 1,994 of the 3,458 DEGs were upregulated at 3 dpi, whereas 1,666 of the 3,031 DEGs were upregulated at 14 dpi (Fig. 2B,C). Among all DEGs, up to 1,768 were commonly found in both time points, including 948 co-upregulated genes and 773 co-downregulated genes (Fig. 2D).

### Overview of citrus transcriptional regulation in response to *D. citri* infection

**Fig. 3:**
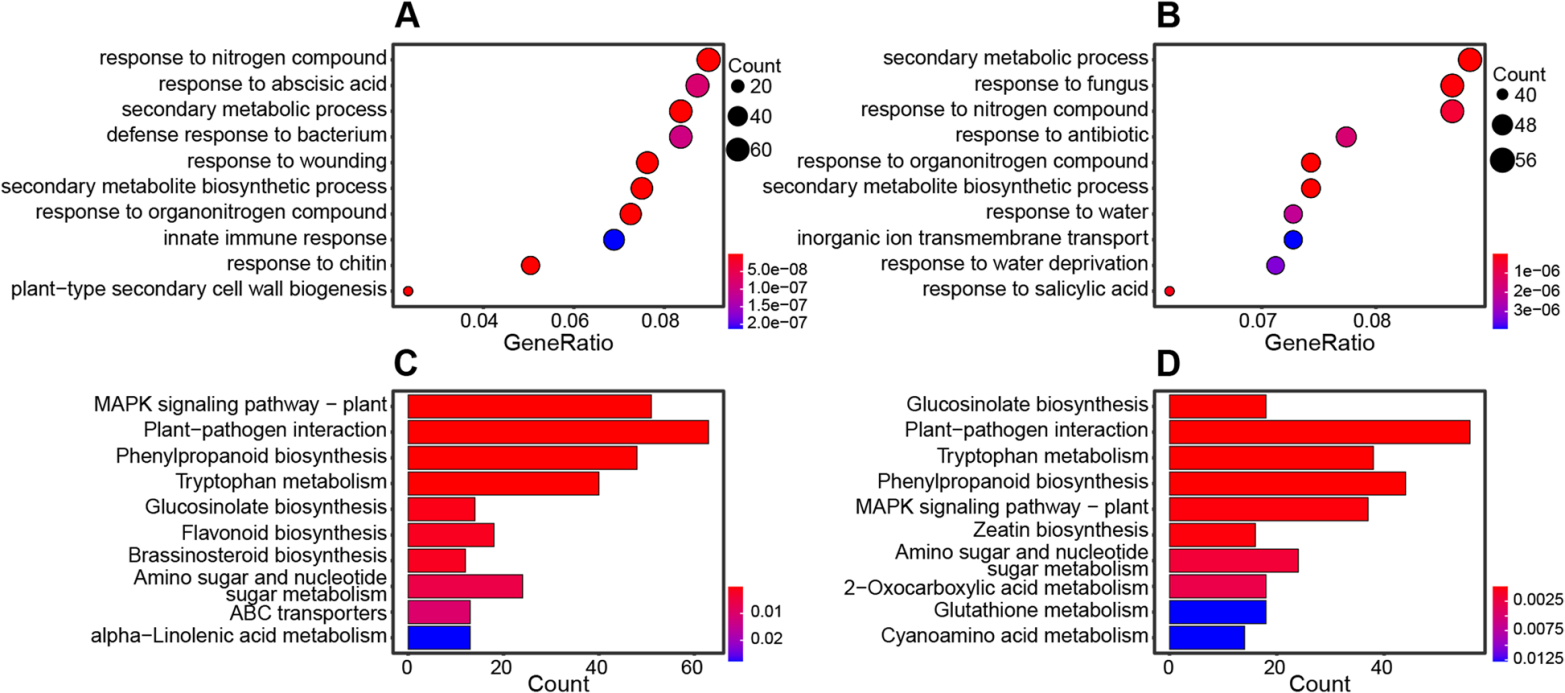
Overview of functional enrichment of citrus leaves in response to *D. citri* infection. Functional enrichment analysis was performed using ClusterProfiler. **A**, **B** Significantly induced GO (gene ontology) categories (top 10 GOs belong to “biological process”) at 3 and 14 dpi, respectively. **C**, **D** Significantly induced KEGG pathways at 3 and 14 dpi, respectively

Functional enrichment analysis was performed for all DEGs using clusterProfiler [14] (adjusted *P* < 0.05), which identified 154 and 210 enriched GO (gene ontology) terms (Fig. 3A, B and Table S3), and 10 and 12 enriched KEGG (kyoto encyclopedia of genes and genomes) pathways, at 3 and 14 dpi, respectively (Fig. 3C, D and Table S4).

There were 97 GO terms enriched at both 3 and 14 dpi, which are mainly involved in response-related biological processes (33 GOs), such as “response to reactive oxygen species”, “immune response”, “response to fungus”, “response to chitin”, “response to wounding”, and “induced systemic resistance”. Several phytohormone response related processes were also found at both 3 and 14 dpi, including “response to ethylene”, “response to abscisic acid”, “response to salicylic acid”, and “response to jasmonic acid”. Moreover, seven KEGG pathways were enriched at both 3 and 14 dpi. Prominent among these was “plant–pathogen interactions” pathway, which comprised the largest number of DEGs over other pathways.

Moreover, 57 GO terms were enriched specifically at 3 dpi, among these the most significantly enriched GO terms were mainly associated with the biosynthesis of plant cell wall, i.e., “plant-type secondary cell wall biogenesis”. In contrast, 113 GO terms were enriched only at 14 dpi, and the significantly enriched GO terms were mainly involved in the plant hormone metabolism and regulation, such as “regulation of hormone levels”, “hormone metabolic process”, and “salicylic acid metabolic process”. In addition, we found that three and five KEGG pathways were enriched only at 3 and 14 dpi, respectively.

### Plant perception and signal transduction upon *D. citri* infection

Recognition is one of the earliest events in plant-pathogen interaction [15]. Plants are able to recognize potential pathogens through host sensors (PRRs, pattern-recognition receptors), and to trigger a series of defense responses called PAMP-triggered immunity (PTI) [16].

**Fig. 4:**
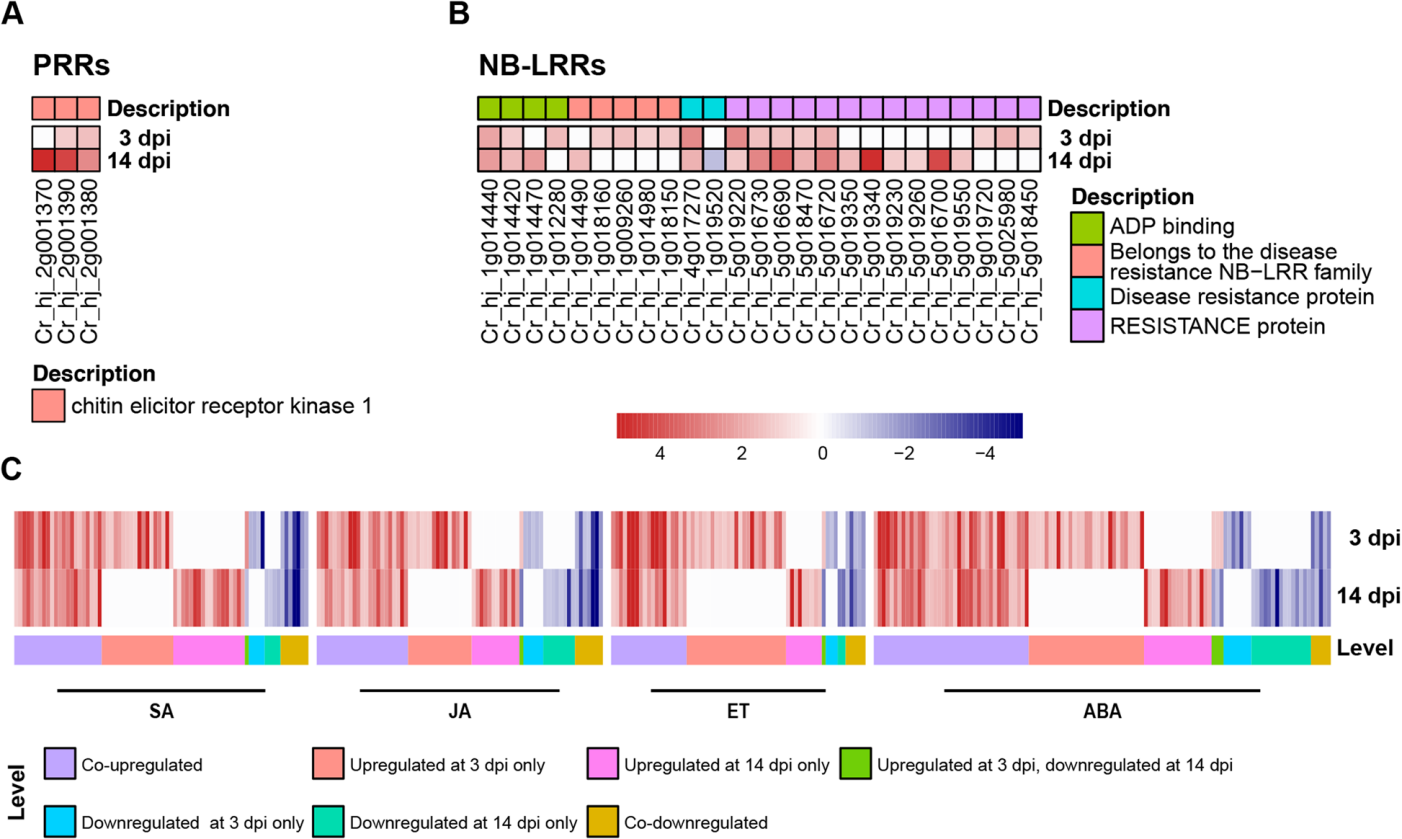
Differential expression of PRRs (pattern recognition receptors), NB-LRR (nucleotide-binding site–leucine-rich repeat protein) and phytohormone genes. Heatmap depicting differential expressed genes of PRRs (**A**), NB-LRR (**B**), and phytohormone (**C**) at 3 and 14 dpi. Differential expressed genes are annotated by eggNOG database. Expression values are presented as log_2_(fold change)

MAPK (Mitogen-activated protein kinase) cascades play central roles in the PTI signaling pathway, transducing signals from PRRs to downstream components for regulation [17]. In this study, the MAPK pathway was significantly induced at both 3 and 14 dpi, which consisted of 51 and 37 DEGs, respectively (Fig. 3C). CERK1 (chitin elicitor receptor kinase 1) is a LysM receptor kinase that belongs to PRRs and is essential for chitin elicitor signaling upon fungal invasion [18]. We observed three significantly upregulated genes encoding CERK1, and two of them were upregulated at both 3 and 14 dpi (Cr_hj_2g001380, Cr_hj_2g001390), while the other one was only upregulated at 14 dpi (Cr_hj_2g001370) (Fig. 4A).

In addition to the front line of innate immune system (i.e., PTI), plants have evolved diverse R proteins, most of which belong to the NB-LRR class, to recognize effector proteins secreted by the pathogens, and subsequently trigger the effector-triggered immunity (ETI) [17]. We found that 25 NB-LRR genes were differentially expressed, most of which (*N* = 24) were upregulated (Fig. 4B). Among these NB-LRR genes, DSC1 (disease resistance-like protein 1), which is known to be able to trigger hypersensitive response in plant defense, showed the highest level of upregulation specifically at 14 dpi.

### Differential expression of phytohormone associated genes

Phytohormones are small molecules produced by plants to regulate diverse physiological processes and plant–pathogen interactions, including plant defense [19]. Genes involved in the biosynthesis and signaling of SA (salicylic acid), JA (jasmonic acid), ET (ethylene), and ABA (abscisic acid) were differentially expressed following *D*. *citri* inoculation. We identified 74 SA-, 72 JA-, 64 ET-, 115 ABA-related DEGs, and most of them were upregulated **(**Fig. 4C**)**.

SA is best known as a defense-related hormone. Plant perception of pathogen attack can induce SA synthesis, leading to activation of plant immunity. NPR1-like gene has been identified as a key component of the SA signaling pathway and is required for plant disease resistance. We observed differential expression of three NPR1-like genes, among which Cr_hj_7g013060 and Cr_hj_7g013090 were only upregulated and downregulated at 3 dpi, respectively, while the other gene (Cr_hj_7g013220) was upregulated at both 3 and 14 dpi. NPR1-like proteins interact with TGACG-binding (TGA) transcription factors and connect with defense response. Here, two TGA2 transcription factors (Cr_hj_5g031660 and Cr_hj_1g005970) were found only upregulated at 3 dpi, which mediated pathogenesis-related (PR) gene expression and disease resistance. Five PR genes were highly expressed, mainly showing upregulation.

ET is a major component of the blend of defense signals that is produced in many plant-pathogen interactions, and functions as an important regulator of plant immunity. Unlike other plant hormones, ET-related DEGs mainly showed upregulation at 3 dpi only (Fig. 4C), suggesting that the ET pathway is possibly involved in the early response of citrus defense. Several genes involved ET-signaling pathway were upregulated (Table S2). The ethylene receptor ETR2, which localized to the ER membrane [20], was upregulated at 3 dpi only (2.1-fold) (Table S2). Concordantly, some regulatory elements downstream of ETR2 were also induced at 3 dpi (Table S2), including the transcription factor EIN3 and defense-related gene CHiB [21]. ET- and JA-signaling often regulate synergistically to activate defense-related genes upon pathogen infection [22] Ethylene response factors (ERFs) act downstream of the intersection between the ET and JA pathways, which are key elements regulating defense response genes [23]. Noteworthy, our data showed that ERFs were upregulated at 3 dpi but downregulated at 14 dpi.

### Cell wall modification of citrus leaves following *D. citri* infection

The germinated *D*. *citri* conidia penetrate the epidermal cell of citrus leaves, which triggers the self-defense reaction of citrus host, leading to formation of a mechanical barricade tissue to inhibit further development of the fungus. The plant cell wall forms a dynamic physical barrier that protects the host against pathogen invasion. Alterations of the cell wall structure may facilitate the restriction of disease development [24, 25].

**Fig. 5:**
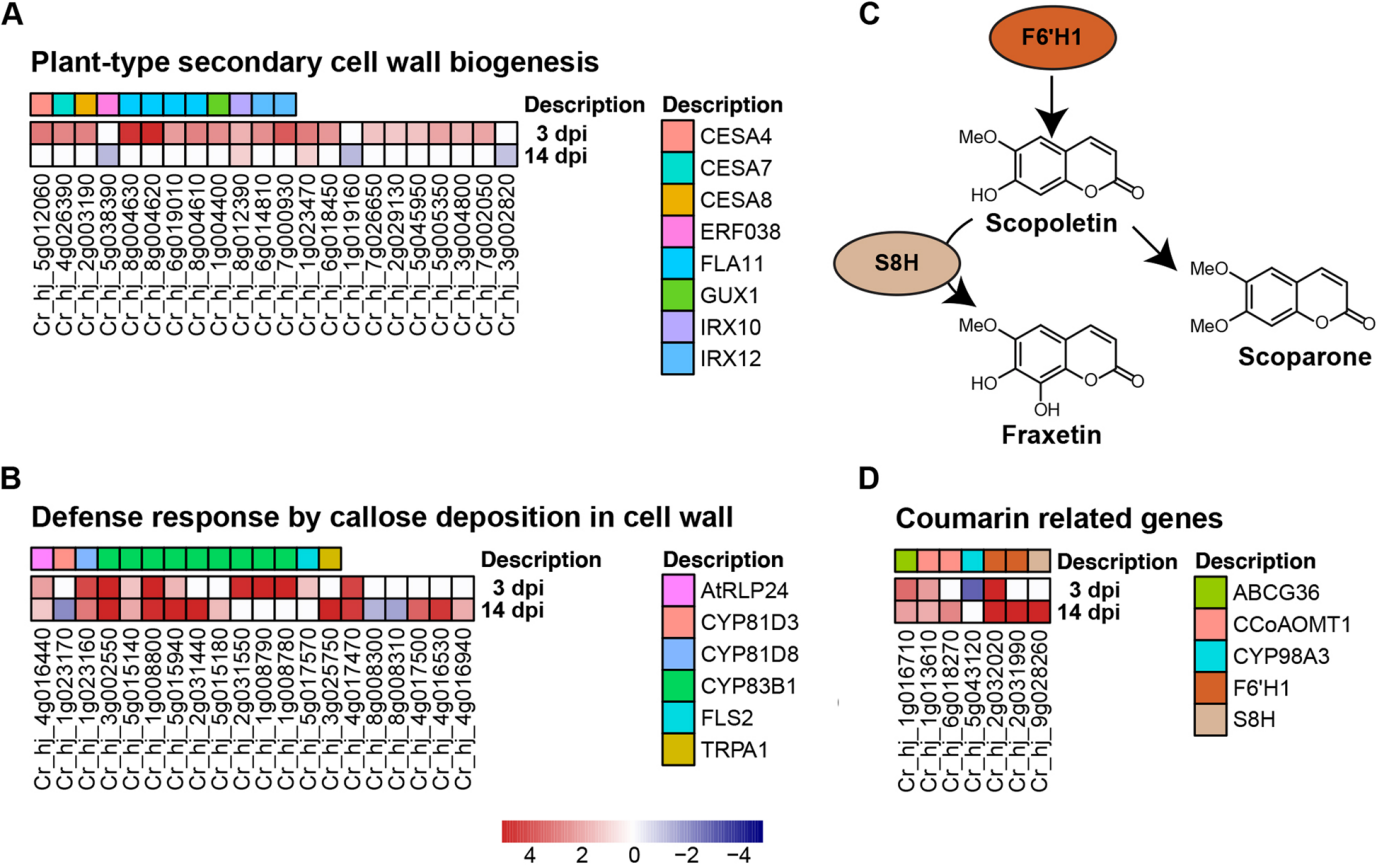
Genes involved in the plant cell wall modification and the coumarin synthesis were effectively activated. Heatmap depicting differentially expressed genes of plant-type secondary cell wall biogenesis (**A**) and defense response by callose deposition in cell wall (**B**). Coumarin biosynthesis pathway (**C**) and the corresponding differentially expressed genes at 3 and 14 dpi (**D**)

Accordingly, we observed that 12 enriched GO terms were associated with the modification of plant cell wall. Remarkably, six of these GO terms were enriched only at 3 dpi and all of them were associated with cell wall biogenesis. Prominent among these was “plant-type secondary cell wall biogenesis” category, including 19 enriched genes (Fig. 5A). These upregulated genes were mainly involved in plant secondary cell wall synthesis, such as FLA11 involved in the initiation of secondary cell wall development, CESA required for cellulose synthesis in secondary cell wall, and IRX10 that is essential for glucuronoxylan biosynthesis.

As a comparison, five of 12 enriched cell wall-related GO terms were specific to 14 dpi, which included the biological process of callose deposition (2 GOs), cell wall thickening (2 GOs) and cell wall modification (1 GOs). Among them, the “Defense response by callose deposition in cell wall” category was the most enriched one, containing 20 DEGs (Fig. 5B). Within the “Defense response by callose deposition in cell wall” category, 11 out of 20 DEGs belonged to the cytochrome P450 family, and 9 were annotated as CYP83B1.

We noted that five DEGs showed highly upregulated expression at 14 dpi (Fig. 2C), while no significant changes at 3 dpi. The pectin methylesterase (Cr_hj_5g001890) was the most highly expressed gene (log_2_FC > 25), whose function is associated with demethylesterification of pectin.

### Coumarin biosynthesis pathways are triggered in infected leaves

In addition to forming a mechanical barrier through the change of cell structure, previous studies have indicated that citrus can produce a phytoalexin (scoparone, 6, 7-dimethoxy coumarin) to inhibit *D*. *citri* infection [12]. Coumarins are ubiquitously found in higher plants where they synthesized in the phenylpropanoid pathway **(**Fig. 5C**)**. Accordingly, KEGG enrichment analysis demonstrated that “phenylpropanoid biosynthesis” pathway was significantly induced in citrus upon *D*. *citri* infection both at 3 and 14 dpi. In this study, we identified seven candidate DEGs associated with coumarin synthesis and regulation **(**Fig. 5D**)**. All of the genes were upregulated at 3 and/or 14 dpi except for one that was downregulated at 3 dpi. Among them, F6’H1 (Feruloyl-CoA 6’-Hydroxylase1) and S8H (scopoletin 8-hydroxylase), which belong to a large enzyme family of the 2-oxoglutarate and Fe^2+^-dependent dioxygenases, were found to be more strongly upregulated at 14 dpi than at 3 dpi. Cr_hj_2g032020, ortholog of F6’H1, was upregulated by 176.1-fold at 3 dpi and by 377.4-fold at 14 dpi. Cr_hj_2g031990, another F6’H1 gene, was upregulated at 14 dpi only (1050.0-fold). Similarly, S8H (Cr_hj_9g028260) was also significantly differentially expressed (243.1-fold upregulated) only at 14 dpi.

### Reactive oxygen species

Cellular H_2_O_2_ is an important reactive oxygen species (ROS) that acts as a signaling molecule regulating various physiological and defense processes [26, 27]. The increasing ROS in plants leads to an oxidative burst that induces cell death and limits further pathogen infection [28]. In this study, the GO term “reactive oxygen species metabolic process” was significantly induced both at 3 and 14 dpi, with 33 and 25 upregulated genes, respectively (Table S3). RBOHD (Respiratory burst oxidase homolog protein D), a calcium-dependent NADPH oxidase that generated superoxide, involves in the ROS generation upon pathogen invasion [29]. We showed that RBOHD was upregulated by 24.8-fold at 3 dpi and by 70.8-fold at 14 dpi (Table S2). Glycolate oxidase (GLO) was able to generate peroxisomal H_2_O_2_ to modulate redox state and induce programmed cell death (PCD) [30]. Four GLOs gene were differentially expressed, with one upregulated at both 3 and 14 dpi (2.6- and 3.0-fold, respectively), and the other three upregulated only at 14 dpi (range from 2.8- to 8.3-fold) (Table S2).

## Discussion

**Fig. 6:**
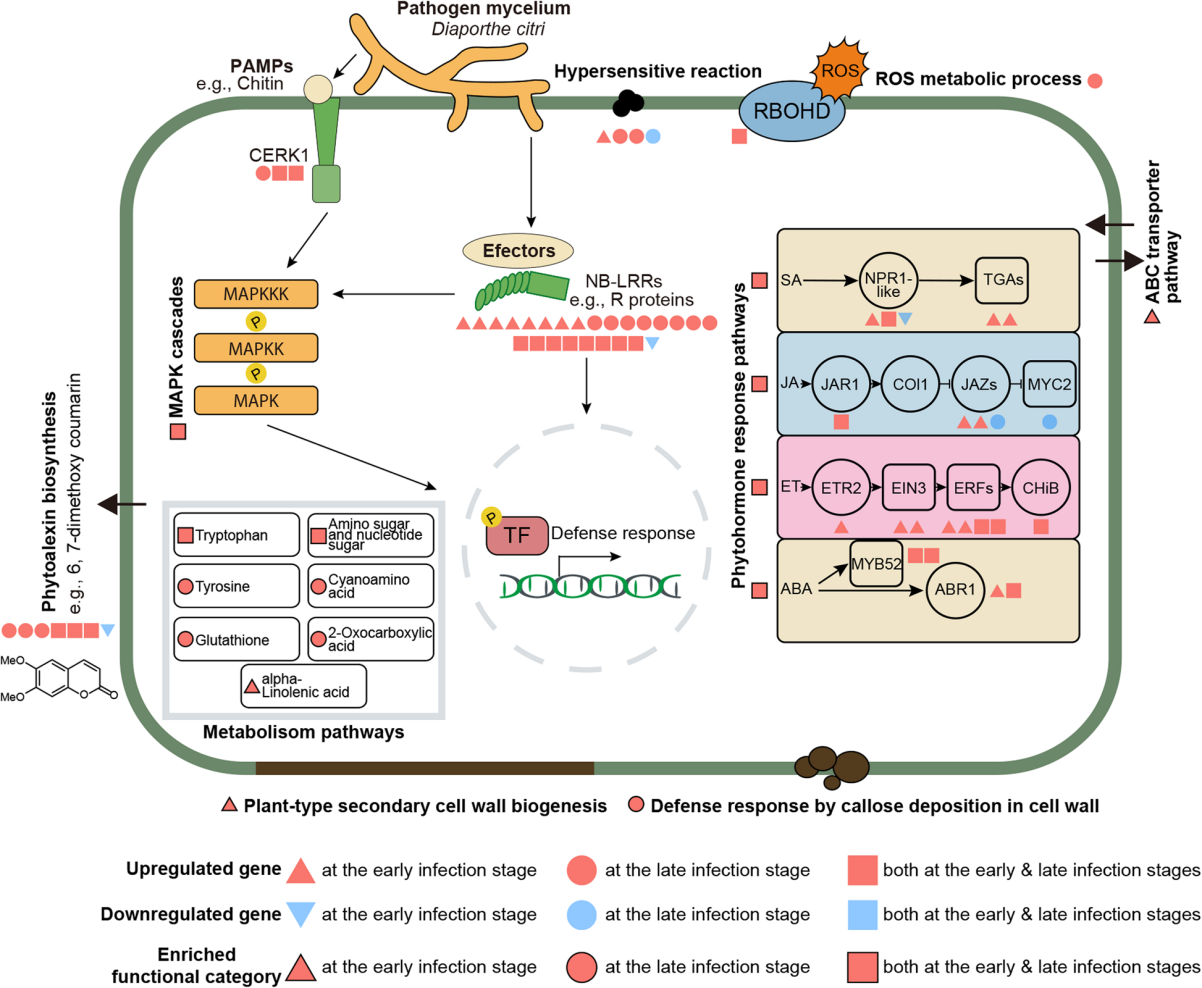
Overview of defense response model against *D*. *citri* in citrus leaves. The early (3 days post inoculation) and late (14 days post inoculation) infection stages show different regulation patterns in response to *D*. *citri* infection. The number of graphics represents the quantity of differentially expressed genes for each category

*D*. *citri* is one of the most destructive fungal pathogens of citrus, which has a worldwide distribution [2]. Previous studies reported that citrus leaves can initiate a series of defense responses to limit *D. citri* invasion and the development of the lesion [10, 12, 31]. However, this finding is based on microscopic examination and HPLC detection, while robust evidence at the molecular level is lacking. In this study, we performed an RNA-seq to profile gene expression in citrus leaves at the early (3 dpi) and late (14 dpi) infection stages upon *D. citri* challenge, and provided molecular insight into the defense response triggered by the fungus **(**Fig. 6**)**.

Plants have been intricately linked with pathogens, and also have evolved several strategies to recognize them [32]. A large number of perception-related genes were induced in citrus leaves following *D. citri* infection, including MAPKs, PRRs, and NB-LRRs. Remarkably, the MAPK cascade pathway is intensively triggered, both at the early and late infection stages. MAPK cascades constitute a network of signaling cascades responsible for multiple defense responses, including defense gene activation, cell wall modification, phytohormone biosynthesis, ROS production and Hypersensitive response (HR) [17]. This suggests that the MAPK cascades is likely to play a central role in the defense response of citrus leaves against *D. citri* infection. More DEGs were found at the early infection stage than at the late infection stage, while more GO terms and KEGG categories were enriched at the late infection stage. This implies that citrus leaves induce more genes of different functional categories participating in the defense response at the late infection stage.

Activation of MAPKs is one of the earliest signaling events for plants to percept PAMPs and pathogen effectors [17]. PTI and ETI were effectively triggered when citrus leaves were attacked by *D. citri*. ROS and HR are downstream components in PTI or ETI, and are associated with pathogen inhibition [33]. ROS have been proposed to act as antimicrobial molecules involved in the reinforcement of plant cell wall and callose deposition to limit the spread of pathogens [34]. We found that ROS biogenesis in citrus leaves was significantly induced at both early and late infection stages. Recognition of effectors by R proteins leads to the activation of ETI, which is often associated with HR at the place of infection. Early responses of the plant defense include pathogen-induced HR, which reduces pathogen penetration and transmission through local plant cell death at the site of infection [32]. In this study, the late infection stage exhibited more stronger expression of R proteins than does the early infection stage. The HR process of citrus leaves against *D. citri* also mainly involved at the late infection stage, possibly mediated by the two highly upregulated genes.

The previous evidence of citrus leaves defending against *D*. *citri* through the self-defense response was from microscopic and scanning electron microscopic (SEM) examinations. This evidence indicated that citrus leaves can limit the expansion of *D*. *citri* mainly through mechanical barriers formed by cell division and coumarin secretion [10, 12, 31]. In the RNA-Seq dataset, we focused on the modification of plant cell wall and the biosynthesis of coumarins. Dissecting the expression of these genes could provide more insight into the self-defense response of citrus leaves against *D*. *citri*.

As the first physical and defensive barrier against pathogens, the plant cell wall usually undergoes dynamic remodeling as an immune response to prevent infection by pathogens [35]. We found that gene expression associated with the plant cell wall exhibited a pattern of temporal regulation. Specifically, plant cell wall biogenesis was mainly induced at the early infection stage, while plant callose deposition response was more active at the late infection stage. This suggests that dynamic cell wall response processes in citrus employ different defense strategies to adapt to *D*. *citri* challenge. In this study, a considerable number of CYP83B1 genes were involved in defense response by callose deposition in cell wall. In *Arabidopsis*, mutant *cyp83B1* was impaired in the induction of the callose response [36]. This suggests that CYP83B1 may play an important role in the defense response of cell wall callose deposition and may contribute to the restricted growth of *D*. *citri*. Remarkably, we observed that a highly upregulated gene at the late infection stage, annotated as PME17, which involves in regulation of pectin demethylesterification. We speculated that citrus leaves were likely to block the further expansion of *D*. *citri* by regulating the demethylesterification of pectin at the late infection stage. This was supported by a previous study, which demonstrated that PME17 triggers pectin methylesterases activity and involves in defense against *Botrytis cinerea* in *Arabidopsis* [37]. Specifically, a greater development of *B*. *cinerea* mycelium was observed around the inoculation site of *pme17* mutant compared to the wild type plant [37].

Coumarins are phytoalexins derived from the phenylpropanoid pathway and are widely found in higher plants [38]. In general, coumarins can be classified into two types, simple (e.g., scopolin, scopoletin and fraxetin) and complex coumarins [39]. Numerous studies have shown that coumarins exhibited antimicrobial activity against plant pathogens, including bacteria, fungi, viruses and oomycetes [39]. Indeed, the extent and timing of coumarin accumulation has often been associated with the level of disease resistance [39]. For instance, the Hevea rubber tree variety resistant to the fungus *Microcyclus ulei* and the oomycete *Phytophthora palmivora* accumulated scopoletin faster and more persistently upon pathogen infection than the susceptible variety [40]. Scopoletin in tobacco is accumulated during a hypersensitive response [41]and is considered to be involved in virus resistance [42]. In citrus, coumarins, such as xanthyletin and scoparone, can act as phytoalexins in resistance to pathogens [43, 44]. Scoparone is the most intensively studied phytoalexin in citrus. Many studies have reported the antagonistic effect of scoparone against pathogens in citrus, such as *Penicillium digitatum*, *P*. *italicum*, *Phytophthora citrophthora*, as well as *D*. *citri* [44]. Citrus leaves were able to produce scoparone upon *D*. *citri* attacking, and restrict the pathogen development [12]. Our RNA-Seq dataset supports the coumarin biosynthesis in citrus leaves against *D*. *citri* and indicates that the coumarin response is stronger at the late infection stage than at the early infection stage. In addition, the S8H gene is also induced (at the late infection stage only) among the coumarin biosynthesis pathway in response to *D*. *citri* infection. However, S8H gene is not directly involved in scoparone synthesis, which implies the production of other coumarins, possibly fraxetin, a coumarin derived from scopoletin [39].

## Conclusions

The differentially expressed genes were analyzed by RNA-Seq analysis between *D*. *citri*- versus mocked samples at 3- and 14-days post inoculation, representing the early and late infection stages, respectively. 1994 out of the 3458 differentially expressed genes were upregulated at the early infection stage, whereas 1666 out of the 3031 differentially expressed genes were upregulated at the late infection stage. Collectively, our study profiled the defense response of citrus leaves against *D*. *citri* infection, involving the coumarin synthesis and plant cell wall modification.

## Methods

### Plant material, *D. citri* inoculation and harvesting

Eight-year-old “Hongjv” cultivar citrus plants were grown in a greenhouse with a 28/20°C day/night temperature regime and natural sunlight at Zhejiang University, located in Hangzhou, Zhejiang Province, China. *D*. *citri* was cultured on potato dextrose agar at 26 °C. To inoculate citrus leaves, conidial suspension of *D*. *citri* was prepared from a 45-day-old culture. The concentration of the conidial suspension was adjusted to 1 × 10^6^ mL^−1^ using a hemacytometer. The young leaves were sprayed with the conidial suspensions. After inoculation, the entire plant was placed in a clear plastic bag sprayed with water for three days to ensure high humidity. The same procedure was followed for the mock inoculation, where distilled water was used instead. Leaf samples were collected at 3- and 14-days post inoculation (dpi) from the pathogen-inoculation and the mock-inoculation treatments. The samples were frozen in liquid nitrogen immediately after collection and kept at -80 °C until use. Five biological replicates were prepared for each time point.

### RNA extraction, library construction and sequencing

RNA was extracted from 100 mg leaf material using the RNeasy Plant Mini kit according to the manufacturer’s instructions (Qiagen, Germany). The quantity and quality of extracted RNA were assessed using the Agilent 2100 Bioanalyzer (Agilent Technologies, USA). RNA-Seq libraries were prepared from 3 µg of each purified RNA with NEBNext UltraTM RNA Library Prep Kit of Illumina (NEB, USA) according to the manufacturer’s instructions. Briefly, the fragmentation of mRNA was enriched from total RNA by Oligo (dT) beads, and double-stranded cDNAs were synthesized. Libraries were purified using the AMPure XP system (Beckman Coulter, USA), and quantified using the Agilent 2100 Bioanalyzer. The pooled libraries were subjected to cluster generation and sequencing was carried out on the Illumina NovaSeq 6000 platform (250-bp, paired-end reads).

### Data processing and analysis

The raw reads were filtered using Trimmomatic [45] to remove adapters and low quality bases. Hisat2 was used for the clean reads to align with the *C. reticulata* reference genome assembly [46]. The read numbers mapped to each gene were counted using featureCounts [47]. Gene expression was calculated using TPM (transcripts per million) normalization. DESeq2 [48] was used to identify differentially expressed genes (DEGs) (adjusted *P* value < 0.05, fold change > 2). DEGs were mapped to the KEGG (Kyoto Encyclopedia of Genes and Genomes) [49] pathways and GO (gene ontology) [50] functional categories using eggNOG-mapper [51]. ClusterProfiler [14] was used to perform GO and KEGG functional enrichment analysis of the DEGs (Benjamini-Hochberg adjusted *P* value < 0.05).

### Supplementary Information

**Additional file 1: Table S1.** Summary statistics of the RNA-Seq data.

**Additional file 2: Table S2.** Differentially expressed genes calculated by DESeq2.

**Additional file 3: Table S3. **Significantly induced GO (gene ontology) categories.

**Additional file 4: Table S4.** Significantly induced KEGG (Kyoto Encyclopedia of Genes and Genomes) pathways.

## Data Availability

Raw RNA-Seq reads have been deposited in the NBCI SRA under the BioProject accession numbers PRJNA734968. The used genome and the gene model information can be found from the following website: http://115.159.76.36/index.html?data=data/hj.
