# Correction: Transcriptional insights of citrus defense response against *Diaporthe citri*

**DOI:** 10.1186/s12870-023-04694-x

**Published:** 2023-12-27

**Authors:** Pudong Li, Xiaoe Xiao, Jingrui Wang, Fan Niu, Jiangnan Huang, Bianyue Xie, Lu Ye, Chaofan Zhang, Dengliang Wang, Qun Wu, Xueliang Zheng, Yunpeng Gai, Hongye Li, Chen Jiao

**Affiliations:** 1 The Key Laboratory of Molecular Biology of Crop Pathogens and Insects of Ministry of Agriculture, The Key Laboratory of Biology of Crop Pathogens and Insects of Zhejiang Province, Institute of Biotechnology, Zhejiang University, Hangzhou, 310058 Zhejiang China; 2 Quzhou Academy of agricultural and Forestry Sciences, Quzhou, 323000 Zhejiang China; 3 Agricultural Characteristic Industry Development Center of Quzhou City, Quzhou, 323000 Zhejiang China; 4 School of Grassland Science, Beijing Forestry University, Beijing, 100083 China

**Correction:** ***BMC Plant Biol*** **23, 614 (2023)**

10.1186/s12870-023-04624-x

Following publication of the original article [1], an error has been spotted in the affiliations of the first two authors, Pudlong Li and Xiaoe Xiao. These authors are affiliated to Aff1 only.

The correction does not affect the overall result or conclusion of the article. The original article has been corrected.
